# Anodal tDCS modulates specific processing codes during conflict monitoring associated with superior and middle frontal cortices

**DOI:** 10.1007/s00429-021-02245-4

**Published:** 2021-03-03

**Authors:** Nico Adelhöfer, Ann-Kathrin Stock, Christian Beste

**Affiliations:** Cognitive Neurophysiology, Faculty of Medicine, Department of Child and Adolescent Psychiatry, TU Dresden, Fetscherstrasse 74, 01307 Dresden, Germany

**Keywords:** Anodal tDCS, EEG, Conflict monitoring, Superior frontal cortex

## Abstract

Conflict monitoring processes are central for cognitive control. Neurophysiological correlates of conflict monitoring (i.e. the N2 ERP) likely represent a mixture of different cognitive processes. Based on theoretical considerations, we hypothesized that effects of anodal tDCS (atDCS) in superior frontal areas affect specific subprocesses in neurophysiological activity during conflict monitoring. To investigate this, young healthy adults performed a Simon task while EEG was recorded. atDCS and sham tDCS were applied in a single-blind, cross-over study design. Using temporal signal decomposition in combination with source localization analyses, we demonstrated that atDCS effects on cognitive control are very specific: the detrimental effect of atDCS on response speed was largest in case of response conflicts. This however only showed in aspects of the decomposed N2 component, reflecting stimulus–response translation processes. In contrast to this, stimulus-related aspects of the N2 as well as purely response-related processes were not modulated by atDCS. EEG source localization analyses revealed that the effect was likely driven by activity modulations in the superior frontal areas, including the supplementary motor cortex (BA6), as well as middle frontal (BA9) and medial frontal areas (BA32). atDCS did not modulate effects of proprioceptive information on hand position, even though this aspect is known to be processed within the same brain areas. Physiological effects of atDCS likely modulate specific aspects of information processing during cognitive control.

**Supplementary Information:**

The online version contains supplementary material available at 10.1007/s00429-021-02245-4.

## Introduction

Conflict monitoring processes have repeatedly been examined (Botvinick et al. 2001; Botvinick 2007) with various experimental paradigms, including Simon tasks (Simon 1990). The common finding in Simon tasks is that responses are slower and more error-prone when an incongruent lateralization of stimulus and responding hand induces a so-called stimulus–response/S-R conflict (Kornblum et al. 1990; De Jong et al. 1994; Keye et al. 2013; Mückschel et al. 2016). In other words, S-R conflicts occur due to an overlap of incongruent stimulus and response features (Kornblum 1994). This suggests that stimulus-related (Hommel 2011) and response-related processes, as well as mapping processes of the stimulus onto the response contribute to the magnitude of conflicts in Simon tasks.

Neurophysiological correlates of conflict monitoring are likely to represent a mixture of these different processes, or processing codes. When assessing event-related potentials (ERPs), Simon conflicts are associated with a larger (more negative) amplitude of the N2 ERP-component, which are most likely caused by activation modulations in the anterior cingulate cortex (ACC), generally important in response monitoring (van Veen and Carter 2002; Botvinick et al. 2004; West et al. 2005; Willemssen et al. 2009; Böckler et al. 2011; Spapé et al. 2011; Shenhav et al. 2013; Clayson and Larson 2013; Stock et al. 2016; Chmielewski and Beste 2017; Beste et al. 2017; Vahid et al. 2020). Even though this finding is very reliable, it is important to consider that most ERP components, including the N2, are composed of various amounts of signals and information from different sources (Nunez et al. 1997; Huster et al. 2015; Stock et al. 2017). The N2 component likely reflects a mixture of different codes related to perceptual processing (‘stimulus codes’) and response-selection (‘response selection codes’) (Folstein and Van Petten 2008; Mückschel et al. 2017). Matching this, medial frontal areas involved in conflict monitoring processes have been suggested to integrate information from different sources to enable or facilitate cognitive control (Cavanagh and Frank 2014; Shenhav et al. 2016).

Of note, it has been suggested that only a subset of the neurophysiological processes involved in conflict monitoring is affected by neurotransmitter-mediated neuromodulation (Mückschel et al. 2017). By integrating data from a temporal EEG signal decomposition approach with pupil diameter data as an indirect index of the norepinephrine system activity, Mückschel et al. (2017) showed that the norepinephrine system selectively modulates response-related conflict monitoring processes in medial frontal areas. In contrast to this, norepinephrine does not seem to modulate stimulus-related codes, even though these are processed at the same time and in adjacent cortical regions (Mückschel et al. 2017). This suggests that neuromodulatory effects on conflict monitoring may be selective for a specific functional sub-process. The norepinephrine system has often been suggested to increase gain control mechanisms (Aston-Jones and Cohen 2005; Nieuwenhuis et al. 2005), associated with better information processing at sensory, cognitive (Servan-Schreiber et al. 1990; Salinas and Thier 2000; Bensmann et al. 2018; Adelhöfer et al. 2018), and motor levels (Greenhouse et al. 2015; Thura and Cisek 2016). However, norepinephrine is by far not the only factor that may influence neuronal gain control by modulating neuronal excitability (Chance et al., 2002; Hay and Segev, 2015; Molaee-Ardekani et al., 2013). Transcranial direct current stimulation (tDCS), for example, affects cortical excitability through the subthreshold modulation of membrane potentials (Stagg and Nitsche 2011; Filmer et al. 2014; Woods et al. 2016; Reinhart et al. 2017). It has recently been shown that the weak direct electric currents boost the gain of synaptic dynamics and co-activation between synaptic inputs (Rahman et al., 2017), which are central for gain control (Servan-Schreiber et al. 1990; Li et al. 2001; Li and Rieckmann 2014). Given that tDCS modulates gain control-relevant processes similarly to norepinephrine (Adelhöfer et al. 2018, 2019), it should also have differential effects on response- and stimulus-related codes. Notably, some recent data provides first evidence for an inter-relation of tDCS effects and effects of the norepinephrine system (Adelhöfer et al. 2019), which substantiates the hypothesis that tDCS effects may be quite similar to effects attributed to neurotransmitter-related neuromodulatory effects. More specifically, we hypothesize that tDCS only modulates processes mapping stimulus to response features, in this case, the selection of the correct response, but not stimulus-related processes, or motor processes during conflict monitoring. This hypothesis seems reasonable considering a Theory of Event coding (TEC) interpretation of the Simon effect (Hommel 2011) and recently results on the neurophysiological underpinnings of TEC (Takacs et al. 2020a, b). It has been argued that the conceptual purity of the Simon task, reflecting the impact of a task-irrelevant stimulus feature (i.e. location) on the particular response, can well be related to the concept of event files (Hommel et al. 2001; Hommel 2009). This concept describes how stimuli are associated/bound to a certain response. Interestingly, signal decomposition methods also used in the current study (see below) have shown that processes involved in mapping stimuli to the response are reflected by event files (Takacs et al. 2020a, b). Therefore, and because conflicts in the present task arise from a mismatch between stimulus and response features, the most likely processing stage of conflict resolution amenable to tDCS modulation is the transition between stimulus and response information.

To investigate this question, we applied anodal (atDCS) before a Simon task was performed with EEG recording in healthy young adults. Anodal tDCS facilitates neuronal excitability (Nitsche and Paulus 2000, 2001) in motor cortices for at least one hour (Nitsche and Paulus 2000; Reinhart and Woodman 2014) in case the longer lasting offline stimulations are applied prior to task presentation. We applied atDCS over the vertex to modulate neural processes in superior frontal structures and supplemental motor areas because these regions are known to play an important role in conflict processing as revealed by EEG source localization studies, fMRI studies and studies using transcranial magnetic stimulation (Rushworth et al. 2004; Nachev et al. 2008; Mars et al. 2009; Stock et al. 2013; Herz et al. 2014; Mückschel et al. 2016). We hypothesize that atDCS applied over the vertex modulates Simon-related conflicts in a rather specific fashion. As a consequence, tDCS effects should be more prominent in incongruent trials than in congruent trials. To dissociate response-related and stimulus-related sub-processes of the N2 component, we applied residue iteration decomposition (RIDE) (Ouyang et al. 2011, 2015a), as already done in previous studies (Mückschel et al. 2017; Chmielewski et al. 2018). While RIDE has been developed to control for intra-individual variability in ERP data (Ouyang et al. 2015a), it can also be used to dissociate different processing codes in the EEG signal, because it decomposes the EEG data into three component clusters with different functional relevance (Ouyang et al. 2015a; Mückschel et al. 2017; Chmielewski et al. 2018): The S-cluster depicts stimulus-related processes, the R-cluster depicts response-related processes (i.e., motor preparation/execution), and the C-cluster depicts central processes that are neither clearly associated with the stimulus or the response. Previously, theoretical considerations stated that stimulus-related and response-selection related aspects of information are coded in the N2 time window (Folstein and Van Petten 2008). Using RIDE, this has already been substantiated by our group for conflict monitoring (Mückschel et al. 2017) and for inhibitory control processes (Chmielewski et al. 2018). Importantly, the stimulus (S)-response (R) translation processes that are central for Simon-conflicts (Hommel 2011) from the perspective of the TEC framework (see above), have been suggested to be mainly reflected by the C-cluster (Verleger et al. 2014, 2017; Bluschke et al. 2017; Ouyang et al. 2017; Mückschel et al. 2017; Wolff et al. 2017; Takacs et al. 2020b), but some proportion of the N2 may also be found in the S-cluster (Folstein and Van Petten 2008; Mückschel et al. 2017). If atDCS applied over the vertex modulates Simon-related conflicts, it is likely that particularly processes related to stimulus–response translation processes are modulated. Therefore, we hypothesize that particularly modulations of atDCS should particularly be reflected in the C-cluster data and not (or much less) in the S-cluster or R-cluster data in the incongruent condition. Given that previous findings suggest that particularly superior frontal areas are involved in (Simon) conflict processing (Rushworth et al. 2004; Nachev et al. 2008; Mars et al. 2009; Stock et al. 2013; Herz et al. 2014; Mückschel et al. 2016), we further hypothesize that atDCS modulations of C-cluster neural activity should be associated with the superior frontal cortex in source localization analyses.

Given that the degree of Simon task S-R conflicts is modulated by proprioceptive information/hand positions (Wiegand and Wascher 2005, 2007; Stock et al. 2013; Stock and Beste 2014; Dharmadhikari et al. 2015), we included a crossed hand condition in our study paradigm. It has been shown that an unusual (crossed) hand position modulates both the Simon effect and demands on conflict monitoring processes (Stock et al. 2013). Interestingly, it has been suggested that this effect is driven by the processing of motor efference copies in the supplemental motor area (SMA) and the middle frontal gyrus (Stock et al. 2013). If our hypothesis was true and atDCS selectively modulates response selection processes reflected by the C-cluster (Verleger et al. 2014, 2017; Bluschke et al. 2017; Ouyang et al. 2017; Mückschel et al. 2017; Wolff et al. 2017), we should not find differential effects of hand position, as this factor is mainly associated with motor processing (Stock et al. 2013). Such a result would provide further experimental evidence that atDCS has very specific effects on a subset of processes involved in the resolution of conflicts.

## Materials and methods

### Participants and power calculation

21 healthy, right-handed participants (mean age 24.6; 14 females) took part in this study. Post hoc power analyses revealed a power of 93.0% (derived using G*Power, type 1-error probability of 0.05, one group, 8 measurements, correlation between repeated measures of 0.5, non-sphericity correction of (1). For this power calculation, we used a medium effect size of 0.251, which was detected in a previous study within a comparable line of research, i.e. investigating 2 mA tDCS effects on neurophysiological measures during cognitive control tasks (Friedrich and Beste 2018). However, the actually obtained neurophysiological effect size in the current study is higher (0.327; see results section). Please also note that dependent measures were registered across many stimulus instances (i.e., trials), which is not considered in the power analysis provided by G*Power. Taken together, the power estimate above can be considered rather conservative.

Participants took part in two experimental sessions, with at least 48 h and a maximum of one week in between the two appointments. All participants had normal or corrected-to-normal vision, no medical or psychiatric diseases, and met safety criteria for atDCS stimulation. All participants were naïve to atDCS experiments and had never taken part in such an experiment, before. All participants were each reimbursed with 30 € after completing both appointments. The study was conducted in accordance with the Declaration of Helsinki. The Ethics committee of the Medical Faculty of the TU Dresden approved of the study. Written informed consent was obtained from all individual participants included in the study.

### Task

**Fig. 1:**
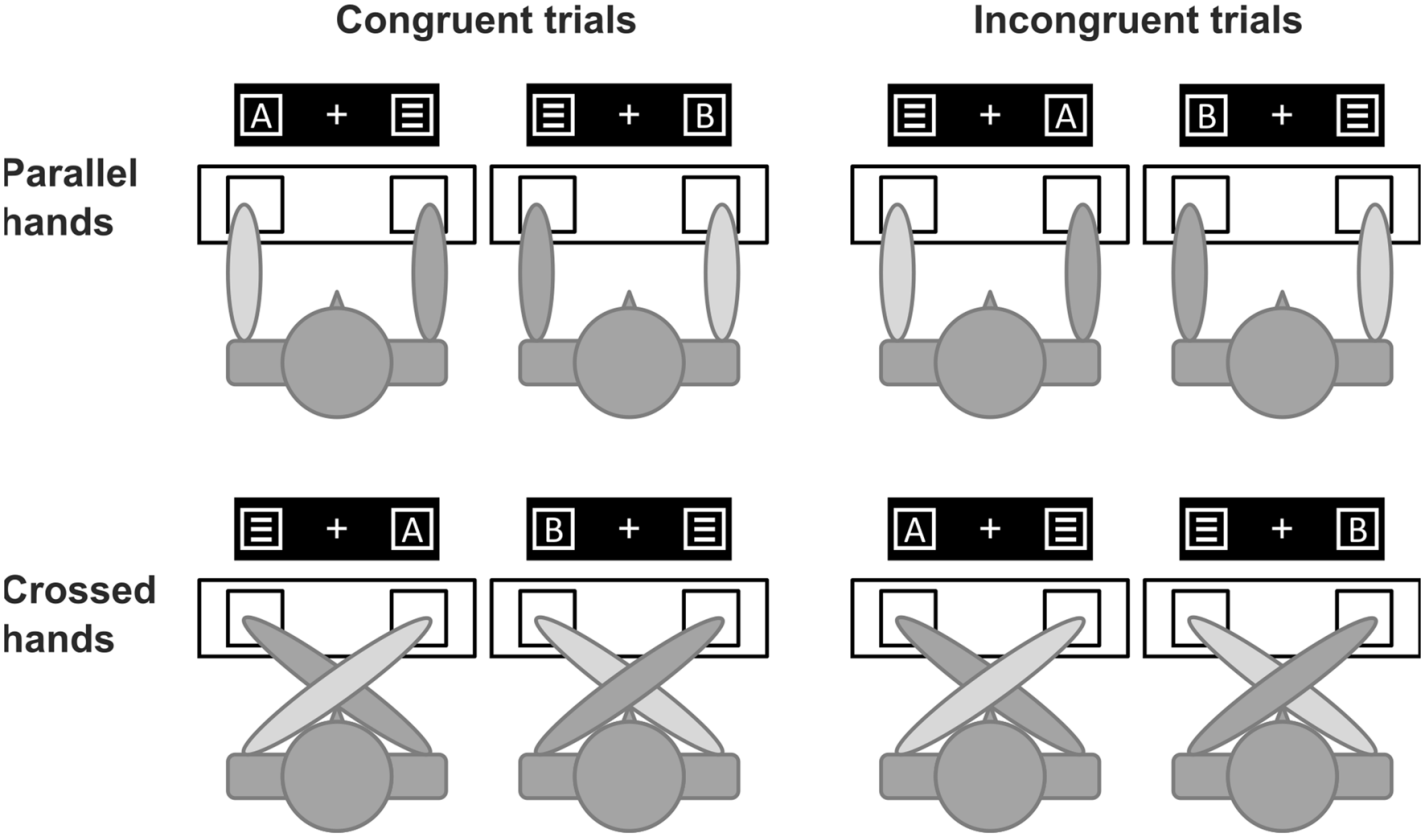
Overview of the different experimental conditions. Hands could be placed on the response buttons in parallel (top row) or crossed, with the left arm above the right arm (bottom row). Target stimulus “A” required responses with the left index finger, while target stimulus “B” required responses with the right index finger (irrespective of whether hands were parallel or crossed). In the illustration, the arm/hand side required for correct responses is highlighted in a lighter gray color than the other arm. The within-subject factor of tDCS stimulation (i.e. tDCS vs. sham) is not displayed. Adapted from (Stock et al. 2013)

This study used a modified Simon task, which has already been used in previous studies of our work group (Dharmadhikari et al. 2015; Zhang et al. 2018) and is illustrated in Fig. 1.

The software Presentation (version 14.9. by Neurobehavioral Systems, Inc.) was used for stimulus presentation, response recording, and sending the EEG triggers. Participants were comfortably seated at a distance of 57 cm in front of a 19″ monitor. In the center of the screen, a white fixation cross and two white frame boxes (1.1° visual angle left and right of the fixation cross) were presented on black background throughout the entire duration of the experiment. Each trial began with a simultaneous presentation of the target stimulus (capital letter A or B) and a contralateral noise stimulus (three horizontal white bars) for 200 ms (see Fig. 1). The left and right “Ctrl” buttons of a regular QWERTZ computer keyboard were used as response buttons and participants were instructed to respond with their left index finger whenever the letter “A” was presented and to respond with their right index finger whenever the letter “B” was presented. Each trial was terminated by the first button press after target onset. Given that delayed responses may affect the Simon effect, a speed-up sign was presented whenever participants failed to respond within 500 ms after target onset. When no response was given, the trial ended after 1700 ms and was coded as “miss”. Response-stimulus intervals (RSI) randomly varied between 2000 and 2500 ms. The experiment consisted of four blocks with 120 pseudo-randomized trials each. In each block, the four possible combinations of target stimulus (A or B) and stimulus position (left or right) occurred equally often. To minimize potential depletion or fatigue effects, the participants were offered to take a break after each experimental block. Hand position varied across blocks to be able to assess the role of proprioception, which may potentially bias the Simon effect (Stock et al. 2013, p. 2, 2015). Participants were asked to place their hands in parallel (i.e. left index finger on the left “Ctrl” button and right index finger on the right “Ctrl” button) in uneven blocks (blocks 1 and 3) and to cross their arms (with the left arm being on the top of the right arm so that the left index finger is on the right “Ctrl” button and the right index finger is on the left “Ctrl” button) in even blocks (blocks 2 and 4). Trials in which the target stimulus and the correct response button were located on the same side were classified as congruent. Trials in which target stimulus and correct response button were located on opposite sides were classified as incongruent. Participants were instructed to respond as fast and as accurately as possible to the stimuli. Datasets were separately aggregated for each subject and appointment following these steps: for each of the four quantified conditions (parallel & congruent; parallel & incongruent; crossed & congruent; crossed & incongruent), we determined the mean response time of all trials (i.e., irrespective of the given response) and also determined the respective standard deviation. We then discarded all single trials, for which the response time was not in the range of mean ± 2 × SD of the respective task condition. This led to the exclusion of 4.5% of all trials (more precisely: sham parallel congruent = 4.3%; sham parallel incongruent = 4.9%; sham crossed congruent = 4.6%; sham crossed incongruent = 4.2%; atDCS parallel congruent = 4.9%; atDCS parallel incongruent = 4.1%; atDCS crossed congruent = 4.6%; atDCS crossed incongruent = 4.1%). All of the examined participants showed sufficiently high hit rates (all above 77%) within any appointment and task condition.

### atDCS protocol

**Fig. 2:**
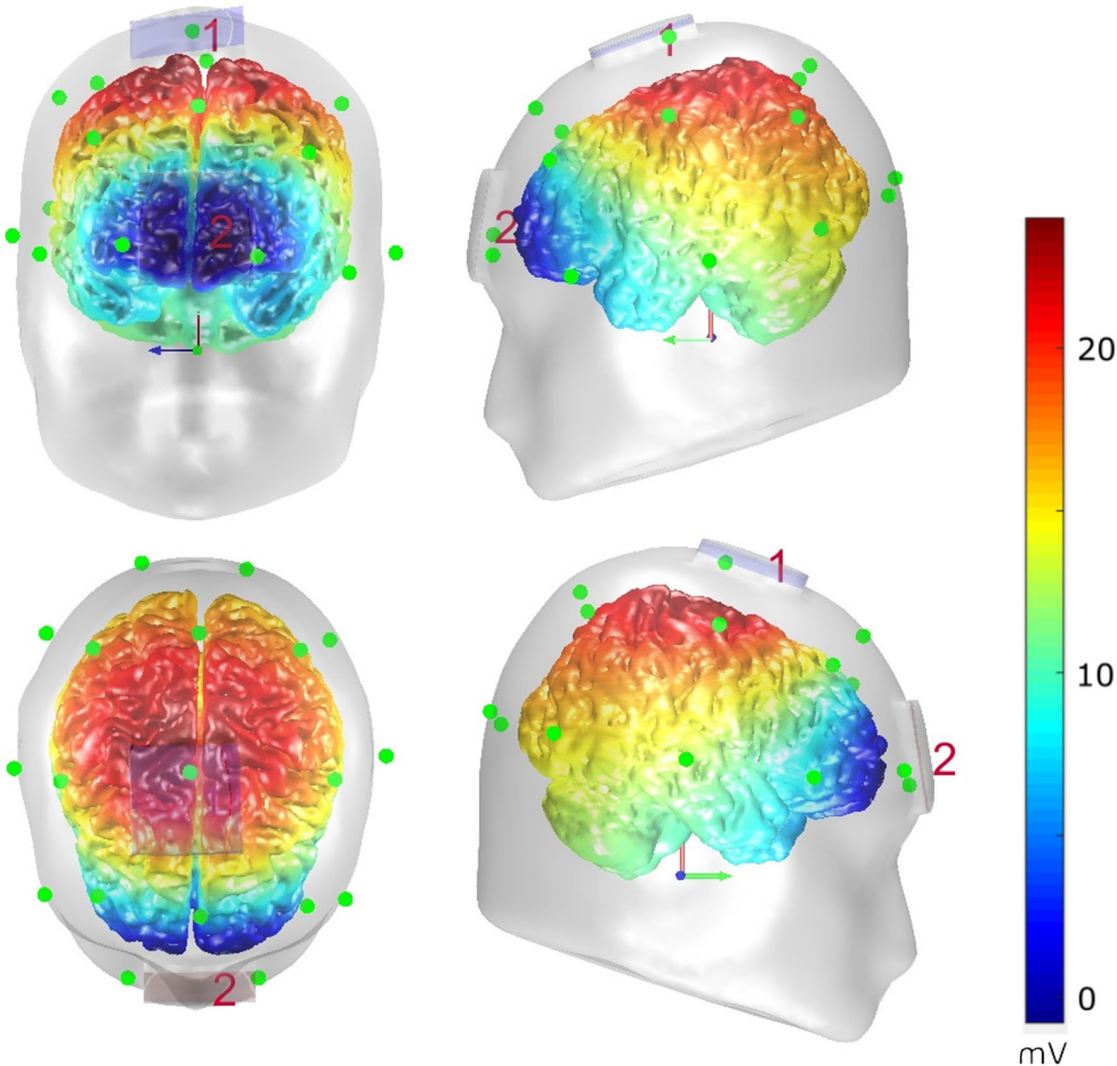
Visual depiction of the electrode setup employed for tDCS. 1: Anode; 2: Reference electrode (cathode). A simulation of the resulting voltage flow (as calculated with the COMETS2 toolbox in Matlab) is shown in color

All participants were naïve to tDCS procedures. Prior to performing the task, participants received 2 mA atDCS (DC-Stimulator Plus; NeuroConn, Illmenau, Germany), which meets tDCS safety criteria (Nitsche et al. 2003; Antal et al. 2017). We used rubber electrodes (5 × 5 cm^2^; NeuroConn, Illmenau, Germany) with Ten20 conductive paste in-between the electrode surface and the skin (paste thickness 5 mm). We employed the same electrode sizes for both active and reference electrode, since a larger reference might induce more skin sensations (Fertonani et al. 2015), which in turn could impede efficient blinding. Electrode impedance levels stayed below 10 kΩ during the whole stimulation duration. The anodal electrode was placed 1.8 cm anterior to electrode position Cz, while the reference electrode was fixated centrally on the forehead using an elastic strap. This setup was chosen to stimulate superior frontal regions including the SMA (Hayduk-Costa et al. 2013; Carter et al. 2015). To investigate stimulation effects, participants received a 16 min of current flow on one of their appointments. During this stimulation, the current linearly increased for 15 s, until it reached its maximum (which was maintained for the full 16 min), and decreased likewise after stimulation. On the other appointment, participants received sham stimulation, during which the maximum current was only applied for the first 30 s. Participants were blind as to the stimulation condition. “Appointment order” (sham first vs. sham second) was counterbalanced across participants and also balanced between the genders. We used the “Comets2” toolbox for MATLAB (Lee et al. 2017) to simulate electrical activity as induced by the tDCS setup. The results of this simulation are shown in Fig. 2.

Please note that immediately after the atDCS protocol was implemented, the EEG cap was prepared for later neurophysiological assessment of atDCS effects during task demands. This procedure took no longer than 10 min given that the cap was prepared by two staff members. Thus, neurophysiological data were assessed in a conservative time window during which atDCS effects could still be expected, given the duration of the stimulation (Kuo and Nitsche 2015).

### EEG recording and analysis

While participants performed the task, EEG data were recorded at 500 Hz by 60 Ag–AgCl electrodes placed in an equidistant setup. Signal amplification was accomplished using a BrainAmp amplifier (Brain Products GmbH). The ground electrode was placed between electrodes Fz, AF3, FC6 and C4 (theta = 58, phi = 78) and the reference electrode was located at Fz (theta = 90, phi = 90). The impedance of all electrodes was kept under 5 kΩ using ‘Nuprep Skin Prep Gel’ and a potassium chloride electrolyte gel. Online EEG recording and offline EEG data processing were performed with Brain Vision Recorder and Brain Vision Analyzer, respectively (both Version 2; Brain Products GmbH). Concerning offline analysis, we first used a bandpass filter (zero phase shift Butterworth; 0.5–18 Hz; 48 dB/oct). Next, all data were re-referenced to the average of all 60 channels before a raw data inspection was performed to identify parts of the EEG that were contaminated by gross technical artefacts. These parts of the EEG were manually rejected before any further analysis steps were performed. Channels that showed no activity were also removed from the EEG and interpolated (on average 1.4 ± 1.6 channels per EEG session) after independent component analysis (ICA, Infomax algorithm). The ICA identified independent components containing blinks, horizontal eye movements, and pulse artefacts. These components were discarded before the backward projection of the data was performed. For the analysis of ERPs, the data were segmented using the onset of the stimulus as the reference time point. The segments lasted from 100 ms pre-stimulus onset to 1300 ms post-stimulus onset, resulting in a total interval length of 1400 ms. Only trials with correct responses were included in the analysis. Subsequently, an automated artefact rejection procedure was performed (maximally allowed voltage step: 50 μV/ms; maximally allowed difference of values in 200 ms intervals: 200 μV; lowest allowed range of activity in 100 ms intervals: 0.5 μV). On average 51, 50, 48, 48, 52, 51, 50 and 50 trials were included for the conditions sham parallel congruent and incongruent, sham crossed congruent and incongruent, atDCS parallel congruent and incongruent and atDCS crossed congruent and incongruent, respectively. These trial numbers allow a reliable quantification of event-related EEG data. These segments were then subjected to a current source density transformation, which results in a reference-free representation of the data and acts as a spatial filter (Nunez and Pilgreen 1991). As a result, the unit of the electrophysiological data is μV/m^2^. In a final pre-processing step, the pre-stimulus baseline was set from −100 to 0 ms before stimulus onset. As a last step, the segments for each condition were separately averaged on the single-subject level. Grand average waveforms were calculated for each condition. These were then used for visual inspection which helped identify relevant electrodes reflecting the different ERP components in our sample. The ERP components were quantified in the time windows and electrode locations detailed in supplemental Table 1. It should be emphasized that the abovementioned choice of electrodes and search intervals was validated with statistical methods (Mückschel et al. 2014). The average amplitude was obtained for all 60 electrodes in each of the mentioned search intervals. To compare each electrode to the average of all other electrodes within a given time interval, Bonferroni correction was applied for multiple comparisons (critical threshold *p* = 0.0007). Only electrodes that had significantly larger mean amplitudes than the other electrodes (i.e., negative for N potentials and positive for P potentials) were selected and kept for analyses. It is important to note that this procedure yielded the same electrodes that we identified during the visual inspection of the data.

**Table 1:** Overview of the different time intervals used to extract mean amplitudes reflecting the listed event-related potentials (ERPs) in the temporally decomposed waveforms (RIDE)

Cluster	ERP component	Electrode	Quantified interval (ms)	Assessed conditions
S	P1	P7, P8	95–115	All
	N1	P7, P8	155–175	All
	N2	FCz	240–270	All
C	N2	FCz	250–270	Congruent trials
			270–305	Incongruent trials
	P3	FCz	355–390	Congruent trials
			385–420	Incongruent trials
R	motor component	C3, C4	380–410	Congruent trials
			430–460	Incongruent trials

### Residue iteration decomposition analysis (RIDE)

For reasons already outlined in the introduction, the EEG data were additionally decomposed into several clusters using the freely available RIDE toolbox (manual available on http://cns.hkbu.edu.hk/RIDE.htm), which provides an established method to do so (Verleger et al. 2014; Ouyang et al. 2015b; Chmielewski et al. 2018). The method is easy to implement given recorded EEG data and can be applied to every EEG data set. The three obtained clusters are time-locked to stimulus-onset (S-cluster), to the response (R-cluster) or reflecting processes that do not temporally correlate with either stimulus presentation or response execution (C-cluster). Since the C-cluster is not closely time-locked to any external event, its latency needs to be determined using an iterative procedure. In short, an initial latency value is determined from a time window function, which is re-estimated based on a template matching approach after the S-cluster is removed in each iteration. This process is repeated until convergence of the initial latency estimation of the S- and C-cluster is reached. More detailed mathematical descriptions can be found in the paper by (Ouyang et al. 2011). The time windows for each cluster need to be pre-specified so that relevant processes are contained. We chose the time windows from −200 to 500 ms and from 100 to 900 ms relative to stimulus onset for the S and C-cluster, respectively. Additionally, we chose the time window from −300 to 900 ms relative to button press for the R-cluster. Importantly, the spatial filter properties of the CSD transformation do not violate the assumptions of RIDE, because scalp distributions are not taken into account by the algorithm (Ouyang et al. 2015b). For statistical analyses, each of the different clusters was quantified at the single-subject level. Electrodes and time windows for data quantification were selected based on a visual inspection of the grand averages of the data (i.e., waveforms separately averaged for each condition across all participants). The electrodes and time windows selected for data quantification are shown in Table 1.

The selected electrodes were again validated using the same statistical procedure as described in the last section.

### Source localization analysis

As significant neurophysiological effects were only obtained after applying RIDE (compare hypotheses and results), we used the RIDE-clusters were for source localization analysis with standardized low resolution brain electromagnetic tomography (sLORETA) (Pascual-Marqui 2002). Of note, a comparable approach has already been taken in a previous study of our group (Chmielewski et al. 2018). Mathematically speaking, the sLORETA algorithm offers a linear solution to the inverse problem without localization bias (Marco-Pallarés et al. 2005; Sekihara et al. 2005). The validity of sources estimated via sLORETA analysis using standard (i.e., not co-registered/measured) electrode coordinates has been corroborated by evidence from fMRI and EEG/TMS-studies (Sekihara et al. 2005; Dippel and Beste 2015). The standardized current density was calculated for each voxel (5 mm edge length, 6239 voxels) in the grid of the standard MNI152 template (Fuchs et al. 2002). We contrasted the sham against the atDCS data using the built-in statistical non-parametric mapping (SnPM) routine. We used voxel-wise randomization tests with 2500 permutations (*p* < 0.01, corrected for multiple comparisons). Voxels with significant differences between sham and stimulation were then plotted in the MNI brain (www.unizh.ch/keyinst/NewLORETA/sLORETA/sLORETA.htm).

### Statistical analysis

Separate repeated-measures ANOVAs were used to analyze the obtained behavioral and neurophysiological data. In all analyses, we used “stimulation” (stim vs. sham), “hand position” (parallel vs. crossed) and “experimental condition” (congruent vs. incongruent stimulus/response locations) as within-subject factors. Whenever necessary, we also used “electrode” as an additional within-subject factor for the neurophysiological analyses. All reported values underwent Greenhouse–Geisser correction and post hoc tests were Bonferroni-corrected, whenever necessary. For all descriptive statistics, the standard error of the mean (SEM) is given as a measure of variability.

## Results

### Behavioral data

Participants gave more correct answers in the parallel (88.8% ± 2.6) than in the crossed hands condition (85.4% ± 3.2) (*F*(1,20) = 20.34; *p* < 0.001; η_p_^2^ = 0.504). The same was true for congruent (92.5% ± 2.4) as compared to incongruent trials (81.6% ± 3.3) (*F*(1,20) = 167.82; *p* < 0.001; η_p_^2^ = 0.894). The ANOVA on hit rates also yielded an interaction effect of hand position × congruency (*F*(1,20) = 4.47; *p* = 0.047; η_p_^2^ = 0.183). Post hoc t tests revealed that there were more correct responses in parallel hands (84.4% ± 1.6) than in crossed hands trials (78.9% ± 1.8) when stimulus and response location were incongruent (*t*(20) = 4.76; *p* < 0.001). However, no such effects of hand position were found for congruent trials (*t*(20) = 1.12; *p* = 0.276). No other main or interaction effects, including the tDCS factor, were found for hit rates (all other *F* ≤ 1.59; *p* ≥ 0.217; η_p_^2^ ≤ 0.111).

**Fig. 3:**
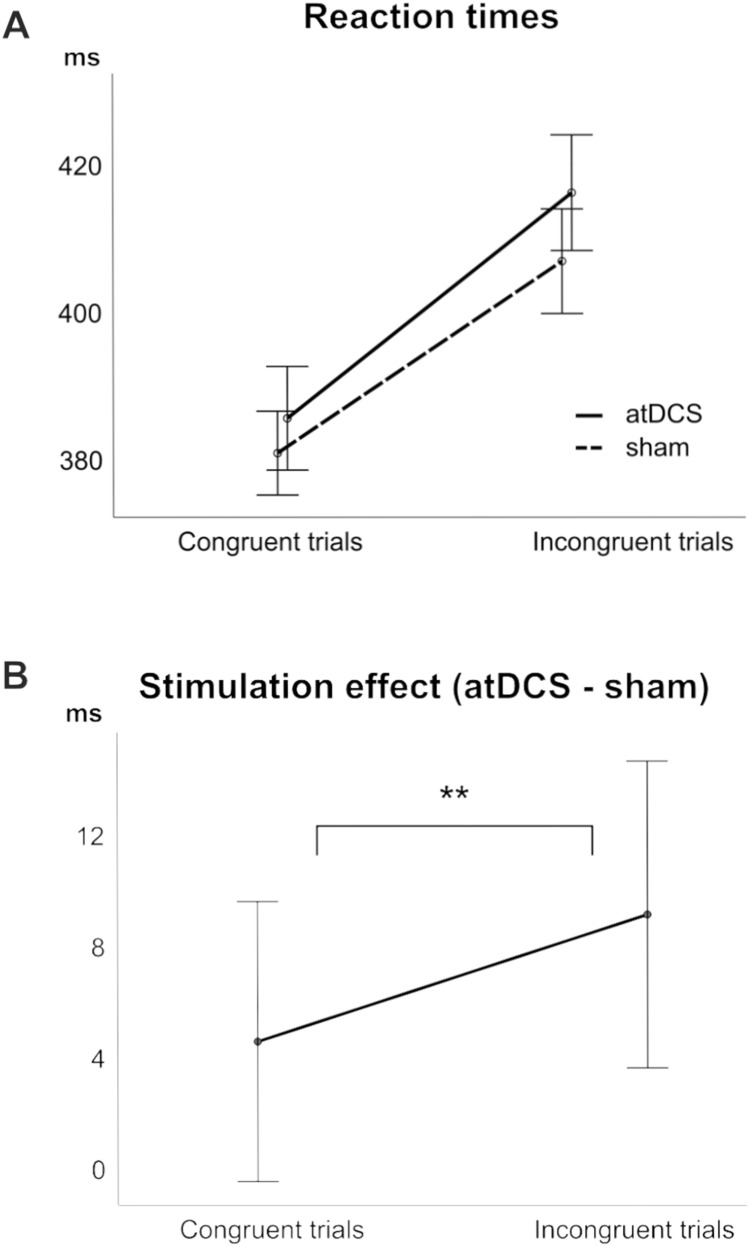
A: Depiction of mean reaction times as a function of stimulus–response congruency (x-axis) and stimulation session (atDCS vs. sham). B: Depiction of the tDCS stimulation effect (obtained by subtracting the sham session from the atDCS session; bottom panel), which was significantly smaller incongruent trials than in incongruent trials. Error bars display the standard error of the mean

The ANOVA on reaction times revealed slower responses in crossed hand trials (404 ms ± 15) than in parallel hand trials (395 ms ± 13) (*F*(1,20) = 11.26; *p* = 0.003; η_p_^2^ = 0.360) as well as slower responses in incongruent (414 ms ± 15) than in congruent trials (385 ms ± 13) (*F*(1,20) = 104.20; *p* < 0.001; η_p_^2^ = 0.839). Importantly, the ANOVA on reaction times also yielded an interaction of tDCS × congruency (*F*(1,20) = 8.60; *p* = 0.008; η_p_^2^ = 0.301), which is illustrated in Fig. 3.

*T *tests revealed that the tDCS effect (i.e., the difference of stimulation minus sham) was larger in incongruent trials (10.14 ms ± 5.71) than in congruent trials (5.33 ms ± 5.24) (*t*(20) = −2.93; *p* = 0.008). More precisely, atDCS significantly increased incongruent trial reaction times compared to sham (stimulation 419 ms ± 8; sham 409 ms ± 7) (*t*(20) = 1.78; *p* = 0.045), which was not the case during congruent trials (*t*(20) = 1.02; *p* = 0.161). No other main or interaction effects were found for response times (all other *F* ≤ 2.04; *p* ≥ 0.169).

Please note that the directions of the reaction time and accuracy effects mismatch. Therefore, the observed increases in response times are likely not due to deliberate response strategies of the participants in favor of response accuracy (in other words, a speed-accuracy trade-off). These results support that participants indeed followed the instructions to respond both as quickly and accurately as possible (refer to Sect. 2.2). Results of the baseline behavioral data (i.e. data including sham sessions only) can be found in the supplemental material.

### Electrophysiological data

#### Non-decomposed event-related potentials

The ERP data obtained from the non-decomposed signal are shown in the supplemental material as our main hypotheses refer to temporally decomposed data. The only finding we would like to point out here is that N2 amplitudes showed a main effect of congruency (*F*(1,20) = 11.99; *p* = 0.002; η_p_^2^ = 0.363) in the expected direction (incongruent trials = −12.03 μV/m^2^ ± 4.36 and congruent trials = −9.46 μV/m^2^ ± 4.20).

#### S-cluster

**Fig. 4:**
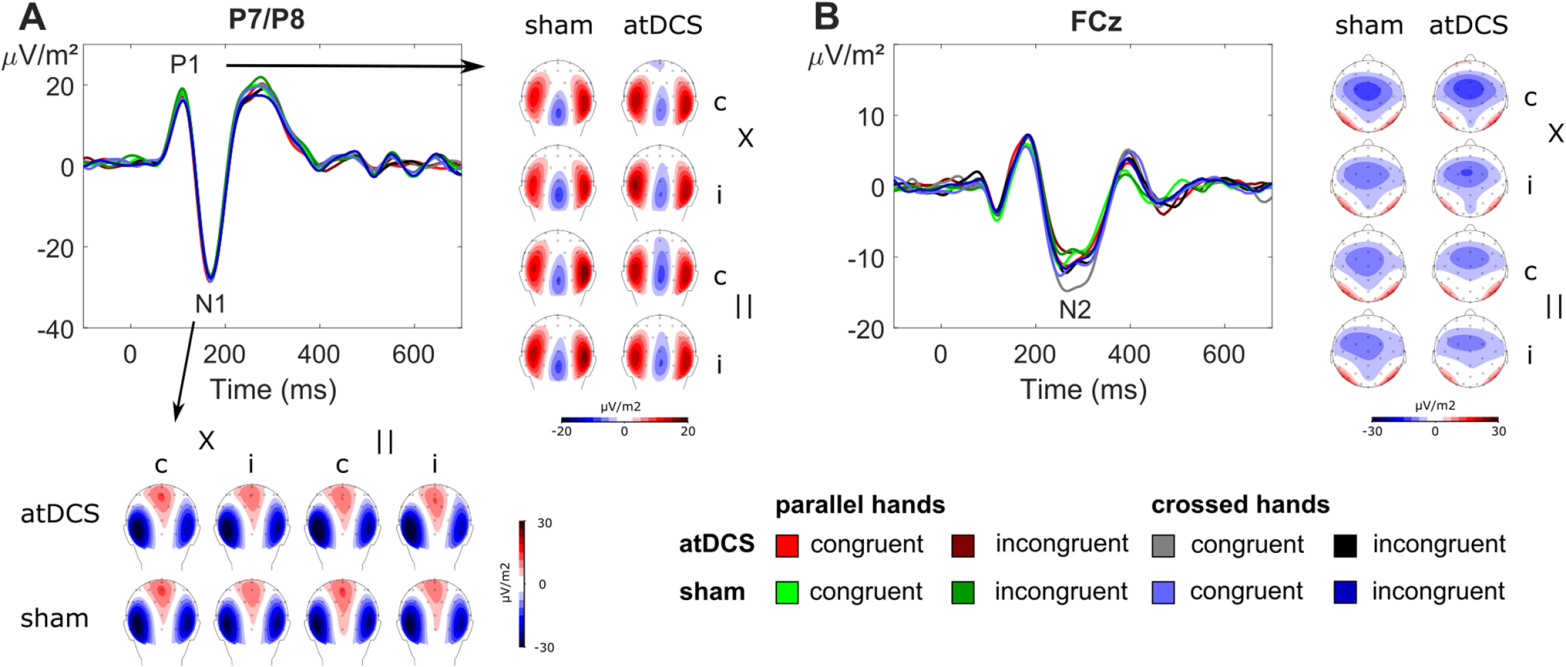
Illustration of the ERP components quantified in the S-cluster with CSD topographies of the respective time windows. Topographies are shown for each combination of experimental conditions, i.e., stimulation session (atDCS vs. sham), stimulus–response congruency (congruent vs. incongruent, denoted “c” and “i”, respectively), and hand position (parallel vs. crossed, denoted “||” and “X”, respectively). A: Average amplitudes of electrode sites P7 and P8 are shown with the P1 time window topographies to the right and the N1 time window topographies at the bottom. C: N2 time window data at electrode site FCz

The results of the S-cluster analyses are depicted in Fig. 4.

In the P1 time window (quantified at electrodes P7 and P8), we found a significant main effect of hand position (*F*(1,20) = 4.72; *p* = 0.042; η_p_^2^ = 0.191), with larger amplitudes in parallel hands (18.14 μV/m^2^ ± 7.07) than in crossed hands (15.73 μV/m^2^ ± 5.79). Additionally, there was an interaction of electrode site × congruency (*F*(1,20) = 8.25; *p* = 0.009; η_p_^2^ = 0.292). Post hoc *t *test showed that electrode P8 showed a significant congruency effect (*t*(20) = −2.27; *p* = 0.034), with larger amplitudes in incongruent trials (18.70 μV/m^2^ ± 4.04) than in congruent trials (17.11 μV/m^2^ ± 3.92 μV/m^2^). Electrode P7 did not show a comparable congruency effect (*t(*20) = 1.60; *p* = 0.126). No other significant main or interaction effects were found for S-cluster P1 amplitudes at the pre-specified electrode sites (all other *F* ≤ 3.75; *p *≥ 0.067).

In the N1 time window (quantified at electrodes P7 and P8), we found an interaction of atDCS × hand position × congruency (*F*(1,20) = 7.86; *p* = 0.011; η_p_^2^ = 0.282). We then conducted separate analyses for each hand position, which revealed a significant interaction effect atDCS × congruency only in the crossed hands (*F*(1,20) = 4.60; *p* = 0.045; η_p_^2^ = 0.187), but not parallel hands condition (*F*(1,20) = 3.81; *p* = 0.065). To further pinpoint this interaction effect, we investigated the stimulation effect (i.e., values measured during the sham session subtracted from values measured during the atDCS session) on amplitudes. After doing so, post hoc *t *tests found a congruency effect in the crossed hands condition, where a larger stimulation effect was found in congruent (2.37 μV/m^2^ ± 2.94) than in incongruent trials (−0.56 μV/m^2^ ± 2.88) (*t*(20) = 2.14; *p* = 0.045). However, there were no significant tDCS effects (stimulation vs. sham) in the congruent crossed hands (*t*(20) = 0.81; *p* = 0.430) or incongruent crossed hands condition (*t*(20) = −0.20; *p* = 0.847). No comparable condition effect was found in the parallel hands condition (*t*(20) = −1.95; *p* = 0.065). Taken together, this pattern of results does not suggest that the atDCS × congruency interaction found for reaction times (see above) is reflected in S-cluster amplitudes in the N1 time window. In other words, the data in the N1 time window cannot explain the behavioral tDCS effects. All other main or interaction effects did not reach statistical significance for S-cluster N1 amplitudes (all other *F* ≤ 4.32; *p* ≥ 0.051).

The ANOVA for the N2 time window (quantified at electrode FCz), yielded a main effect of hand position (*F*(1,20) = 8.44; *p* = 0.009; η_p_^2^ = 0.297), with larger amplitudes in crossed hands (−12.71 μV/m^2^ ± 3.35) than in parallel hands (−9.35 μV/m^2^ ± 3.01). There were no other main or interaction effects for S-cluster N2 amplitudes (all other *F* ≤ 1.78; *p* ≥ 0.198).

#### C-cluster

**Fig. 5:**
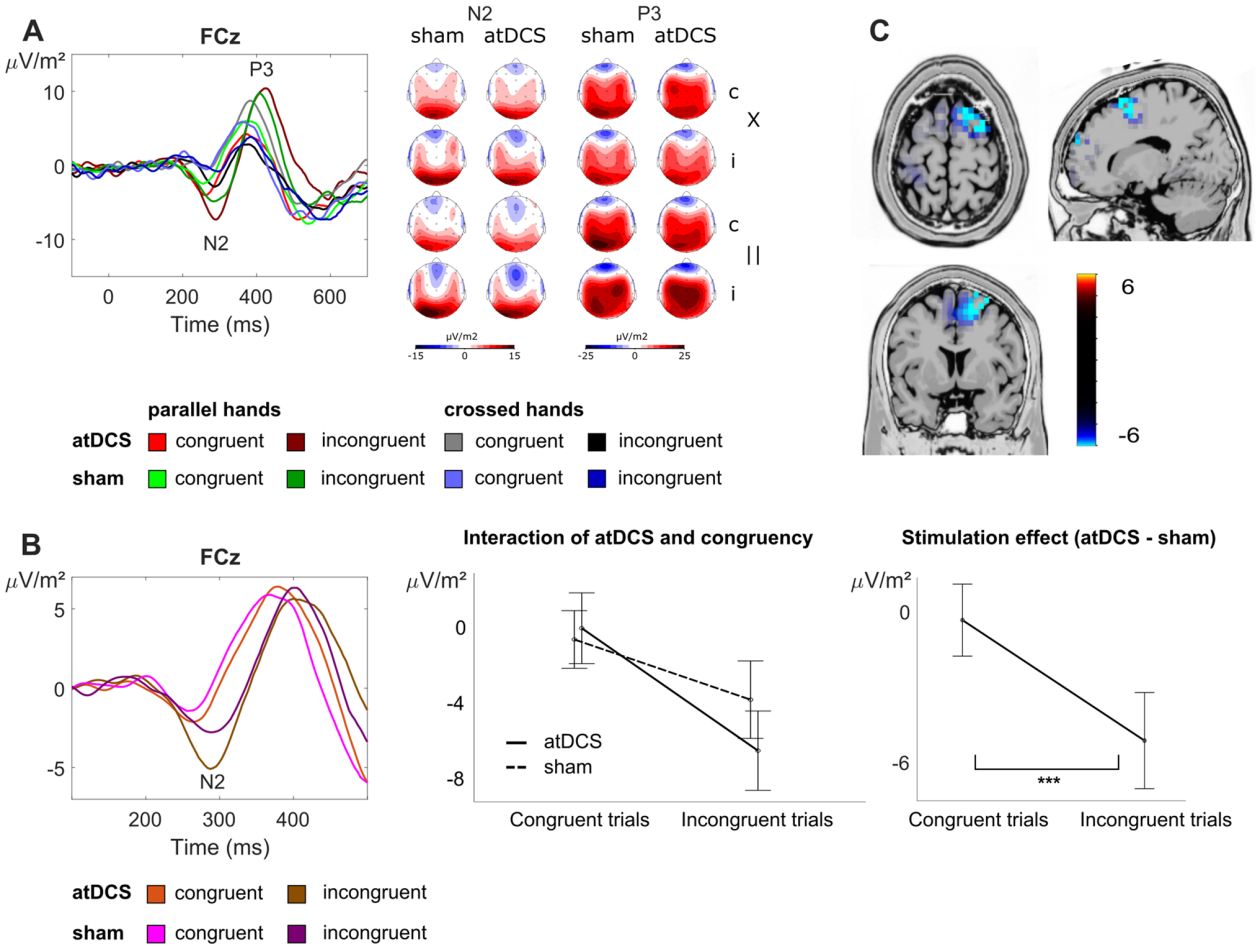
Illustration of the ERP components quantified in the C-cluster. A: Left: Grand average data at electrode site FCz with depictions of the N2 and P3 time windows. Right: CSD topographies for each combination of experimental conditions, i.e., stimulation session (atDCS vs. sham), stimulus–response congruency (congruent vs. incongruent, denoted “c” and “i”, respectively), and hand position (parallel vs. crossed, denoted “||” and “X”, respectively) for the respective time windows (N2 and P3). B: Clearer depiction of the interaction effect found in the N2 time window: The left panel shows mean amplitudes that have been averaged over both hand positions. The middle panel displays C-cluster N2 amplitudes as a function of stimulus–response congruency (x-axis) and stimulation session (atDCS vs. sham). The right panel depicts the tDCS stimulation effect (obtained by subtracting the sham session from the tDCS session). The tDCS effect was significantly larger in the incongruent condition. Note the close resemblance to the interaction effect shown for response times in Fig. 3. Error bars display the standard error of the mean. C: sLORETA results of this interaction effect suggest the medial and middle frontal gyri are associated with this effect

The results of the C-cluster analyses are illustrated in Fig. 5.

The N2 amplitudes (quantified at electrode FCz) yielded a main effect of hand position (*F*(1,20) = 10.28; *p* = 0.004; η_p_^2^ = 0.340), with larger amplitudes in parallel hands (−4.85 μV/m^2^ ± 3.78) than in crossed hands trials (0.68 μV/m^2^ ± 3.93). A main effect of congruency (*F*(1,20) = 18.91; *p* < 0.001; η_p_^2^ = 0.486) revealed larger amplitudes in incongruent trials (−4.41 μV/m^2^ ± 4.05) as compared to congruent trials (0.24 μV/m^2^ ± 3.07). Importantly, there was also an interaction of tDCS × congruency (*F*(1,20) = 9.70; *p* = 0.005; η_p_^2^ = 0.327). Matching the results found for response time data (see above), post hoc *t *tests revealed a larger atDCS effect (i.e., the difference of stimulation minus sham) for incongruent trials (-3.40 μV/m^2^ ± 1.44) than for congruent trials (1.45 μV/m^2^ ± 1.69) (*t*(20) = 3.11; *p* = 0.005). In the source localization analysis using sLORETA, we contrasted the stimulation effect (atDCS—sham) between congruent and incongruent trials. This revealed that the (right) superior frontal gyrus (BA6) was associated with the observed effects. The sources furthermore extended in to middle frontal (BA32) and medial frontal regions (BA9). No other main or interaction effects were found for the C-cluster N2 amplitudes (all other *F* ≤ 1.05; *p* ≥ 0.318).

For the P3 time window (quantified at electrode FCz), there was an interaction of hand position × congruency (*F*(1,20) = 7.34; *p* = 0.014; η_p_^2^ = 0.268). Post hoc *t *tests revealed that in crossed hands yielded larger amplitudes (8.39 μV/m^2^ ± 3.15) than parallel hands (4.89 μV/m^2^ ± 2.82) in congruent trials (*t*(20) =−2.41; *p* = 0.026). No such effect was found in incongruent trials (*t*(20) = 1.67; *p* = 0.111). There were no other main or interaction effects for the C-cluster P3 amplitudes (all other *F* ≤ 1.02; *p* ≥ 0.325).

#### R-cluster

**Fig. 6:**
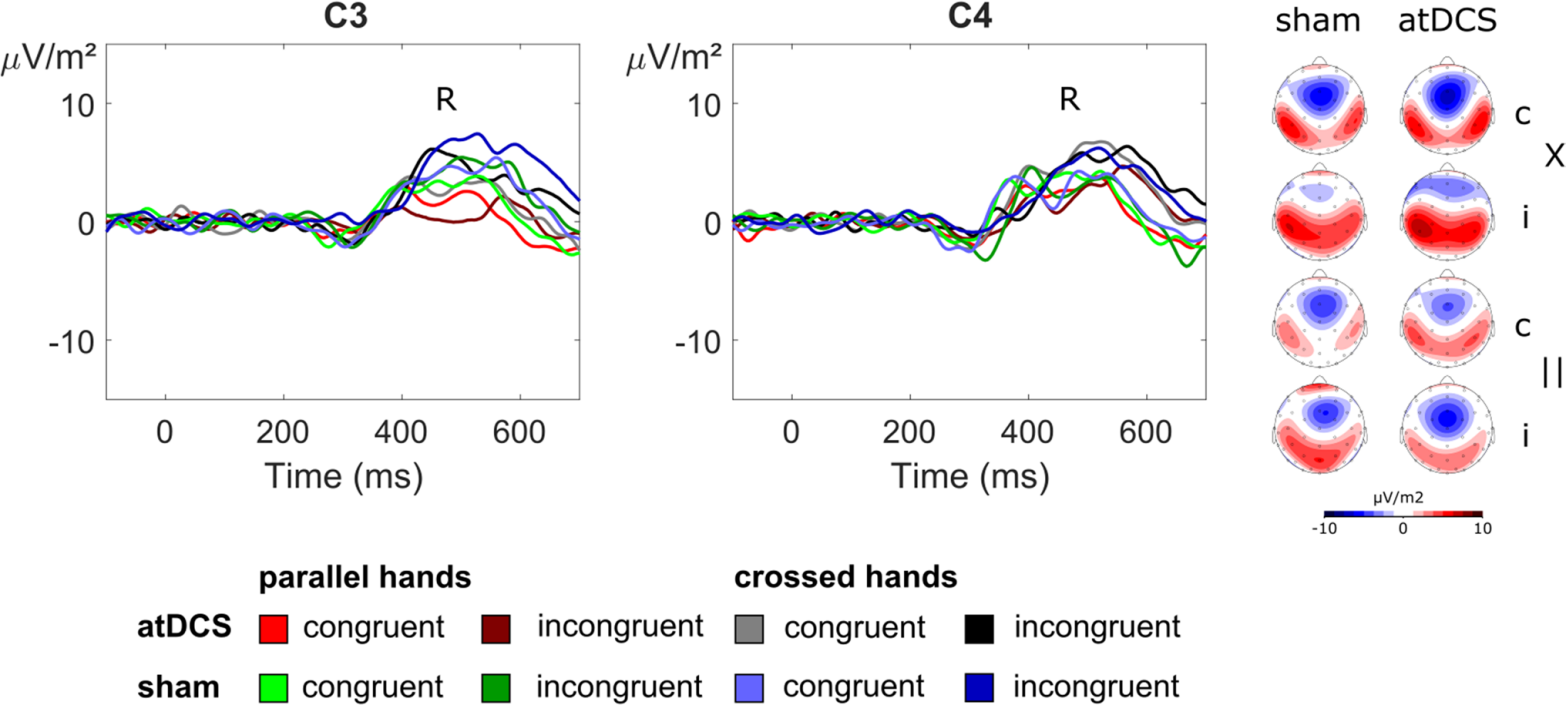
Illustration of the ERP components quantified in the R-cluster at electrode sites C3 and C4, with CSD topographies of the relevant time window (labeled “R”; see Table 1 for details). Topographies are shown for each combination of experimental conditions, i.e., stimulation session (atDCS vs. sham), stimulus–response congruency (congruent vs. incongruent, denoted “c” and “i”, respectively) and hand position (parallel vs. crossed, denoted “||” and “X”, respectively)

The results of the R-cluster analyses are depicted in Fig. 6.

We measured amplitudes at the time point of the motor response/button press at electrodes C3 and C4, as this approach was suggested by visual inspection and corroborated using statistical means (see methods section for details). The statistical analysis of the obtained amplitudes yielded a main effect of hand position (*F*(1,20) = 7.38; *p* = 0.013; η_p_^2^ = 0.269), with larger amplitudes in crossed hands (6.32 μV/m^2^ ± 2.21) than in parallel hands (3.55 μV/m^2^ ± 2.50). There was also an interaction effect of electrode × hand position (*F*(1,20) = 39.84; *p* < 0.001; η_p_^2^ = 0.666). To investigate this interaction effect further, we calculated the hand position effect (parallel minus crossed hands condition). Post hoc *t *tests found that electrode C3 was characterized by larger amplitudes in the parallel hand condition than the crossed hands condition (8.23 μV/m^2^ ± 2.35), while the opposite was true for electrode C4 (–13.76 μV/m^2^ ± 1.62) (*t*(20) = 6.31; *p* < 0.001). No other significant main or interaction effects were found in the R-cluster (all other *F* ≤ 4.15; *p* ≥ 0.055).

#### Additional Bayesian analyses

The above results suggest a substantially stronger effect of atDCS for the incongruent condition, as compared to the congruent condition, that was not modulated by hand position. At the neurophysiological level, the behavioral findings were only mirrored by N2 amplitudes quantified in the C-cluster. To substantiate the specificity of these effects (i.e., the lack of interactive hand position effects), we conducted additional Bayesian analyses using the method of Masson (2011). With this method, the probability of the null hypothesis being true, given the obtained data *p*(*H*_0_/*D*) can be calculated. The results of this analysis can be found in Table 2.

**Table 2:** Bayesian probabilities (i.e., probabilities of the null and alternative hypotheses, given the obtained data) for the interactions, tDCS × hand position, tDCS × hand position × congruency and tDCS × congruency in behavioral and electrophysiological data. Please note that in all but one case, probabilities clearly favor the null hypothesis (i.e., the absence of this interactive effect), given both the behavioral data and the temporally decomposed EEG data (RIDE)

Data	tDCS × hand position		tDCS × hand position × congruency		tDCS × congruency	
	P(H0|D)	P(H1|D)	P(H0|D)	P(H1|D)	P(H0|D)	P(H1|D)
Reaction times	0.694	0.306	0.816	0.184	0.097	0.903
Hit rates	0.811	0.189	0.820	0.180	0.813	0.187
ERP P1 time window	0.670	0.330	0.452	0.548	0.820	0.180
ERP N1 time window	0.402	0.598	0.084	0.916	0.789	0.211
ERP N2 time window	0.792	0.208	0.766	0.234	0.799	0.201
ERP P3 time window	0.807	0.193	0.717	0.283	0.821	0.179
RIDE: S-Cluster P1 time window	0.788	0.212	0.817	0.183	0.820	0.180
RIDE: S-Cluster N1 time window	0.721	0.279	0.124	0.876	0.820	0.180
RIDE: S-Cluster N2 time window	0.652	0.348	0.738	0.262	0.752	0.248
RIDE: C-Cluster N2 time window	0.797	0.203	0.804	0.196	0.067	0.933
RIDE: C-Cluster P3 time window	0.817	0.183	0.805	0.195	0.807	0.193
RIDE: R-Cluster	0.820	0.180	0.812	0.188	0.818	0.182

*ERP* event-related potential, *RIDE* residue iteration decomposition

Of note, the null hypothesis is more likely to be true than the alternative hypothesis when *p*(*H*_0_/*D*) exceeds 0.5, which equals a 50% probability (Raftery 1995). Taken together, the results of the Bayesian analysis provide stronger evidence for the null hypothesis than for the alternative hypothesis for all behavioral and neurophysiological parameters, except for N2 amplitudes in the C-cluster.

## Discussion

In the current study, we examined the effects of atDCS on conflict monitoring processes. In this context, we were particularly interested in whether atDCS has selective effects on different cognitive sub-processes/information coded in neurophysiological signals. To this end, we applied a temporal EEG signal decomposition method in combination with source localization analyses to examine the effect of atDCS.

Contrary to our hypothesis, atDCS over the vertex specifically induced larger conflict (congruency) effects, as atDCS effects were larger in incongruent than in congruent trials. The findings that atDCS increased response times in incongruent trials suggests that it aggravated conflict monitoring and resolution processes; i.e., became slower during conflict monitoring but did not change in accuracy. Thus, atDCS leads to the unexpected paradoxical effect of inducing larger conflict (congruency) effects.

The RIDE-decomposed EEG data provide further insights into the possible reasons for this. A study by Zmigrod et al. (2016) found no modulations of Simon conflicts after stimulation of dorsolateral prefrontal regions. Related to the current findings, the entire pattern of results suggest that atDCS modulation of S-R conflicts differs between functional neuroanatomical regions in the prefrontal cortex. The obtained data may be regarded to be at odds with several recent tACS studies revealing performance increases upon theta-band stimulation over comparable stimulation sites during S-R conflicts (van Driel et al. 2015; Fusco et al. 2018; Lehr et al. 2019; Giller et al. 2020). However, it needs to be noted that tACS entrains oscillatory activity (Herrmann et al. 2016) while tDCS modulates different mechanisms based on the membrane potential (Stagg and Nitsche 2011; Filmer et al. 2014; Woods et al. 2016; Reinhart et al. 2017). Therefore, the tACS and atDCS results are not directly comparable.

Importantly, the neurophysiological data showed that atDCS effects are not only determined by the exact neuroanatomical region in the prefrontal cortex, but are selective for a specific fraction coded in the neurophysiological signal—the C-cluster. In line with this, we did not find atDCS effects in the classical N2 component (refer supplemental material). The N2 component is an established correlate of Simon congruency effects (van Veen and Carter 2002; Botvinick et al. 2004; West et al. 2005; Böckler et al. 2011; Spapé et al. 2011; Shenhav et al. 2013; Clayson and Larson 2013; Stock et al. 2016; Chmielewski and Beste 2017; Beste et al. 2017), but reflects a mixture of perceptual sub-processes and response-selection sub-processes (Folstein and Van Petten 2008; Mückschel et al. 2017). While we also found interaction effects including the atDCS factor in the S-cluster N1 time window, these could not be attributed to significant effects of atDCS in the condition shown in the behavioral data. Therefore, the neurophysiological effect pattern points to a specific modulatory effect of atDCS in the C-cluster N2 time window. This specificity/dissociation was further underlined by the Bayesian analysis of the data, which provides strong support for a lack of atDCS effects in any other time windows and RIDE-clusters (i.e., the S cluster and the R-cluster). Thus, atDCS does not exert a general effect on neural processes involved in conflict monitoring. It seems that atDCS specifically facilitates the resolution of stimulus–response conflicts specifically by enhancing cognitive sub-processes associated with stimulus–response translation processes, which have consistently been associated with the C-cluster (Verleger et al. 2014, 2017; Ouyang et al. 2017; Takacs et al. 2020b). The higher N2 C-cluster amplitude in incongruent than congruent trials is completely in line with the literature (Clayson and Larson 2013) and that a higher conflict effect is associated with increased N2 amplitudes. The simulation of electrical activity induced by atDCS (refer Fig. 2) suggested that we should have modulated superior and middle frontal areas. Matching this, source localization procedures revealed that atDCS effects were associated with superior frontal areas including the supplementary motor cortex (SMA, BA6), as well as middle frontal (BA9) and medial frontal areas (BA32). This fits to data showing that these regions are involved in the resolution of conflicts and stimulus–response translation processes (Rushworth et al. 2004; Nachev et al. 2008; Mars et al. 2009; Stock et al. 2013; Herz et al. 2014; Mückschel et al. 2016). Supporting the interpretation that only specific processes are susceptible to modulations by atDCS in superior frontal regions, the SMA has been shown to process motor-related aspects (i.e., hand position/proprioception) that contribute to the Simon effect (Stock et al. 2013). Given that this manipulation, known to modulate Simon effect size (Wiegand and Wascher 2005, 2007; Stock et al. 2013; Stock and Beste 2014; Dharmadhikari et al. 2015), was unaltered by atDCS (as demonstrated by Bayesian analyses), it seems that atDCS does not modulate motor-related processes during S-R conflict processing, which are most likely depicted by the R-cluster. Thus, it seems that atDCS specifically modulates some fraction of information being processed in superior frontal regions. The question is why atDCS may have increased a higher level of conflict, compared to sham stimulation? At the moment we can provide only some theoretical explanations:

The facilitatory effect of atDCS has mostly been described in motor cortices (Nitsche and Paulus 2000) and in other cortical region paradoxical effects can occur especially when it comes to cognitive control processes (Friedrich and Beste 2018). Another explanation could be that atDCS strengthen the cognitive representations of stimulus–response associations in the event file, as suggested be the TEC account of the Simon task (Hommel 2011). Indeed, the C-cluster reflects representations stimulus–response associations (Takacs et al. 2020a, b) in time windows also relevant for this study. Simon effects have been supposed to be explainable be reconfiguration processes of such stimulus–response associations in incongruent trials (Hommel 2011). Therefore, it is well conceivable that atDCS has strengthened automated stimulus–response representations in the Simon task, which, especially in incongruent trials, lead to declines in task performance. This may have happened by affecting gain modulation principles. Gain control has traditionally been brought into connection with actions of the norepinephrine system (Aston-Jones and Cohen 2005; Nieuwenhuis et al. 2005) and it is known that electric currents boost the gain of synaptic dynamics and co-activation between synaptic inputs (Rahman et al. 2017), which is relevant for gain control (Servan-Schreiber et al. 1990; Li et al. 2001; Li and Rieckmann 2014). More recently, it has been suggested that effects of the norepinephrine system and atDCS are commutable (Adelhöfer et al. 2019). From that perspective the results may be interpreted that different aspects of information coded during conflict monitoring are differentially susceptible to physiological effects induced by brain stimulation. Of note, the study by Adelhöfer et al. (2019) examined atDCS on inhibitory control processes. Inhibitory control processes play an important role in the resolution of response conflicts (Stürmer et al. 2000; Cisek and Kalaska 2005; Taylor et al. 2007; Verleger et al. 2009; Tandonnet et al. 2011; Ocklenburg et al. 2011; Klein et al. 2014). It may, therefore, be speculated that the observed specificity of atDCS effects is due to the involvement of inhibitory control processes (but see (Thunberg et al. 2020) reporting no effects of frontal tDCS on inhibitory control).

Future studies may investigate whether the stimulation of other target regions in prefrontal and parietal cortices may also reveal similar specific effects. The restriction to one cortical region is a limitation of the current study. Further research needs to be conducted to elucidate the mediating physiological mechanisms explaining these differences in greater detail. This will then also provide more insights whether there is a role of inhibitory control in atDCS effects as discussed above.

In summary, the current study examined the effects of atDCS over superior frontal regions on neurophysiological subprocesses involved in conflict control. We demonstrated very specific effects. The effect of superior frontal atDCS on behavior was largest in case of response conflicts and only stimulus–response translation/response-selection processes were affected. This was only visible after decomposing the EEG data. In contrast to this, entirely stimulus-related processes as well as purely response-related processes were not modulated by superior frontal atDCS. This effect was likely driven by activity modulations in the superior frontal areas, including the supplementary motor cortex (SMA, BA6), as well as middle frontal (BA9) and medial frontal areas (BA32).

## Supplementary Information

Below is the link to the electronic supplementary material.

Supplementary file1 (DOCX 408 KB)

## Data Availability

Data will be made available upon reasonable request.

## Code availability

Published software package were used and indicated in the methods section. Scripts for data analysis will be made available upon reasonable request.
