# Supplementary material for: Anodal tDCS modulates specific processing codes during conflict monitoring associated with superior and middle frontal cortices
Source: Brain Struct Funct. 2021 Mar 3;226(4):1335–51. doi: 10.1007/s00429-021-02245-4 (PMC8036188; doi:10.1007/s00429-021-02245-4)
Supplement: Supplementary file 1 — Supplementary file1 (DOCX 408 KB) [file 429_2021_2245_MOESM1_ESM.docx]

**aSupplemental Material**

**Anodal tDCS modulates specific processing codes during conflict monitoring in superior and middle frontal cortices**

Nico Adelhöfer, Ann-Kathrin Stock, Christian Beste

*Supplemental Table 1*

Overview of the different time intervals used to extract mean amplitudes reflecting the listed event-related potentials (ERPs). All time values are relative to stimulus onset.

| **ERP component** | **Electrode(s)** | **Quantified time interval (in ms)** | **Assessed conditions** |
| --- | --- | --- | --- |
| P1 | P7, P8 | 95 - 120 | All |
| N1 | P7, P8 | 155 - 175 | All |
| N2 | FCz | 280 - 300 | All |
| P3 | Cz | 380 - 410 | All |

*Baseline behavioral data*

We found two main accuracy effects in the sham session data. First, participants responded more accurately in the parallel hands (88% ± 1.5) compared to the crossed hands condition (84% ± 2.1) (F(1,20) = 6.46; p = .019; η_p_^2­^ = .244). Furthermore, response accuracies were higher in congruent (91% ± 1.6) compared to incongruent trials (81% ± 2.0) (F(1,20) = 175.55; p < .001; η_p_^2­^ = .898). The interaction effect hand position × congruency was not significant (F(1,20) = 2.60; p = .123). The sham session reaction time data reveal a main effect of congruency (F(1,20) = 75.83; p < .001; η_p_^2­^ = .791) with shorter reaction times in congruent (382 ms ± 6) compared to incongruent trials (409 ms ± 7). No other main or interaction effects in sham reaction time data were found (all F(1,20) ≤ 3.48; p ≥ .077).

*Non-decomposed event-related potentials*

The non-decomposed ERPs can be found in supplementary figure S1.


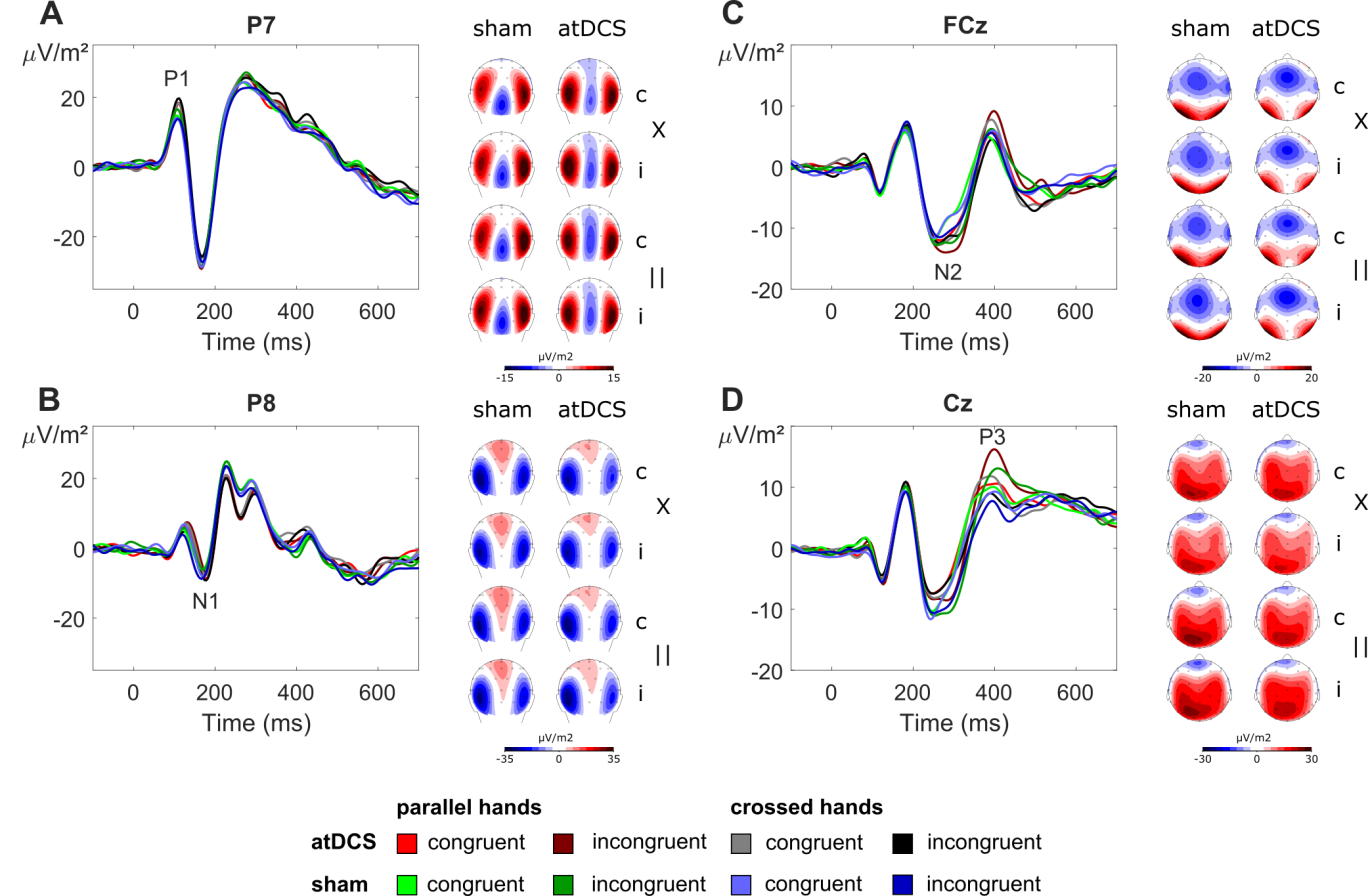


*Figure S1*

Illustration of the ERP components quantified in non-decomposed EEG data, with CSD topographies of the respective time windows always shown on the right side of each graph. Topographies are shown for each combination of experimental conditions, i.e., stimulation session (atDCS vs. sham), stimulus-response congruency (congruent vs. incongruent, denoted “c” and “i”, respectively), and hand position (parallel vs. crossed, denoted “||” and “X”, respectively). A: P1 component at electrode P7. B: N1 component at electrode P8. C: N2 component at electrode FCz. D: P3 component at electrode Cz.

For P1 amplitudes (quantified at electrodes P7 and P8), the ANOVA revealed a significant main effect of tDCS (F(1,20) = 16.02; p = .001; η_p_^2^ = .445), with larger amplitudes during tDCS sessions (16.48 μV/m^2^ ± 5.93) than during sham sessions (8.39 μV/m^2^ ± 5.25). There was also an interaction of electrode × tDCS (F(1,20) = 8.74; p = .008; η_p_^2^ = .304). Post hoc t-tests revealed that the tDCS effect (i.e., the difference of stimulation minus sham) was larger at electrode P8 (12.88 μV/m^2^ ± 3.06) than P7 (3.29 μV/m^2^ ± 2.02) (t(20) = -2.96; p = .008). Hence, the data suggest a lateralized, right-hemispheric tDCS effect on early attentional stimulus processing (Luck et al., 2000). No other main or interaction effects of P1 amplitudes reached statistical significance (all other F ≤ 3.90; p ≥ .062).

For N1 amplitudes (quantified at electrodes P7 and P8), the ANOVA revealed a main effect of electrode site (F(1,20) = 34.08; p < .001; η_p_^2^ = .630), with larger amplitudes at P7 (-27.68 μV/m^2^ ± 6.79) than at P8 (-15.98 μV/m^2^ ± 7.69). There was also a main effect of tDCS (F(1,20) = 11.18; p = .003; η_p_^2^ = .359), with larger amplitudes during the atDCS session (-26.29 μV/m^2^ ± 8.45) than during the sham session (-17.37 μV/m^2^ ± 6.37). In addition, we found an interaction effect of electrode site × tDCS (F(1,20) = 13.01; p = .002; η_p_^2^ = .394). Post hoc t-tests showed larger amplitudes during atDCS (15.46 μV/m^2^ ± 3.43) than during sham stimulation (2.58 μV/m^2^ ± 3.97) at electrode site P8 (t(20) = 4.22; p < .001) but not P7 (t(20) = 1.62; p = .120). In addition, higher amplitudes were measured at electrode site P7 (14.21 μV/m^2^ ± 2.70) than P8 (2.58 μV/m^2^ ± 3.97) during the sham condition (t(20) = 2.55; p = .019), but not during the atDCS session (t(20) = .70; p = .492). We also found interactions of electrode site × hand position (F(1,20) = 7.86; p = .011; η_p_^2^ = .282) and electrode site × hand position × congruency (F(1,20) = 4.42; p = .048; η_p_^2^ = .181). This effect did however not survive post hoc analyses. Finally and most importantly, we also found an interaction of tDCS × hand position × congruency (F(1,20) = 9.04; p = .007; η_p_^2^ = .311). Investigating each hand position in separate post hoc ANOVAs, we found an interaction of tDCS × congruency in parallel hands (F(1,20) = 7.12; p = .015; η_p_^2^ = .263), with larger tDCS effects in congruent trials (-11.39 ± 2.55 μV/m^2^) than in incongruent trials (-8.83 ± 2.64 μV/m^2^) (t(20) = -2.67; p = .015). In crossed hands, there was no such interaction of tDCS × congruency (F(1,20) = 3.01; p = .089; η_p_^2^ = .131). No other main or interaction effects were found for N1 amplitudes (all other F ≤ 4.02; p ≥ .059).

Importantly, the N2 amplitude (quantified at electrode FCz) showed effects of both task manipulations, which are well in line with previous findings on the Simon effect (see introduction): There was a main effect of congruency (F(1,20) = 11.99; p = .002; η_p_^2^ = .363), with larger amplitudes in incongruent trials (-12.03 μV/m^2^ ± 4.36) than in congruent trials (-9.46 μV/m^2^ ± 4.20). In addition, an interaction of hand position × congruency was found (F(1,20) = 5.93; p = .024; η_p_^2^ = .220). Post hoc t-test showed that in parallel hands, N2 amplitudes were larger in incongruent trials (-13.17 μV/m^2^ ± 2.20) than in congruent trials (-9.10 ± 2.14 μV/m^2^) (t(20) = 3.83; p = .001). This congruency effect could not be found in crossed hands (t(20) = 1.29; p = .213). No other main or interaction effects were found for N2 amplitudes (all other F ≤ 2.20; p ≥ .153).

For the P3 amplitudes (quantified at electrode Cz), the ANOVA yielded a main effect of hand position (F(1,20) = 9.48; p = .006; η_p_^2^ = .321), with larger amplitudes in parallel hands (13.14 μV/m^2^ ± 6.42) than in crossed hands (9.67 μV/m^2^ ± 6.36). There was also an interaction of hand position × congruency (F(1,20) = 19.22; p < .001; η_p_^2^ = .490). Post hoc t-tests revealed that incongruent trials resulted in larger P3 amplitudes (15.19 μV/m^2^ ± 3.21) than congruent trials (11.09 μV/m^2^ ± 3.09) in case of parallel hands (t(20) = -3.16; p = .005) but not in case of crossed hands (t(20) = 2.02; p = .057). Likewise, parallel hands yielded larger P3 amplitudes (15.19 μV/m^2^ ± 3.21) than crossed hands (8.40 μV/m^2^ ± 3.20) in incongruent trials (t(20) = 4.37; p < .001), but not in congruent trials (t(20) = .14; p = .893). No other main or interaction effects were found for P3 amplitudes (all other F ≤ 2.43; p ≥ .135).

*Exploratory bivariate correlations between behavior and neurophysiology*

As described in the main text, both neurophysiology and behavior was jointly modulated by atDCS and task demands. More precisely, N2 amplitudes in the C cluster mirrored the reaction time effects: Both measures showed a congruency effect based on the tDCS effect (i.e., stimulation minus sham).

In order to test whether there is a clear linear relationship between these behavioral and neurophysiological measures, we derived the variables accordingly (incongruent trial tDCS effect minus congruent trial tDCS effect) for both reaction time and C cluster N2 data. Bivariate Pearson correlations show no significant correlation (r = -.023; p = .920). Therefore, this data can not conclusively show that there is a linear relationship between these two measures. This question should be revisited using larger sample sizes. Furthermore, at present it is not clear which function best describes the relationship between these measures, which should be the target of future investigations.
